# Association between reserve, white matter integrity and cognitive function in Taiwan community‐dwelling older adults

**DOI:** 10.1002/alz.084829

**Published:** 2025-01-09

**Authors:** Tzu‐Chi Ou, Yi‐Fang Chuang

**Affiliations:** ^1^ National Yang Ming Chiao Tung University, Taipei Taiwan; ^2^ National Yang Ming Chiao Tung University, Taipei, NA Taiwan

## Abstract

**Background:**

The benefit of the reserve, including brain and cognitive reserve, on cognitive functions has been examined in several studies. However, there has been limited evidence about the underlying mechanism, particularly in relation to white matter (WM) integrity. This study aims to investigate the influence of brain and cognitive reserve on cognition and white matter tract integrity among community‐dwelling non‐demented older adults.

**Method:**

We included 379 non‐demented community‐dwelling older adults (287 cognitively normal and 92 mild cognitive impairment [MCI]). The sum of weighted education years and occupational skill level (International Standard Classification of Occupations 2008, ISCO‐08) was used to construct cognitive reserve (CR). The intra‐cranial volume (ICV) was obtained from brain MRI images and used as a proxy of brain reserve (BR). Cognition was assessed across five domains: global cognition, memory, attention, executive function, and language. Fractional anisotropy (FA) values were derived from diffusion tension imaging (DTI), indicating WM integrity. The FA of the global tract was the average of eight major bilateral WM tracts (Fornix, Cingulum, Uncinate fasciculus [UF], Corona radiata, Superior longitudinal fasciculus, Inferior frontal occipital fasciculus, Corpus callosum, Posterior thalamic radiation). We also measured medial temporal lobe (MTL) tracts, the mean from three bilateral MTL WM tracts (Hippocampal cingulum, Fornix, and UF).

**Result:**

The mean age of the study population is 67.7 (±7.2). CR had positive effects on all cognitive domains in the total sample; the effects were stronger on memory (*partial r* = 0.39, *p* <0.001) and attention (*partial r* = 0.53, *p* <0.001) in normal older adults and stronger on global cognition (*partial r* = 0.55, *p* <0.001) and attention (*partial r* = 0.56, *p* <0.001) in MCI; BR only had positive effects on attention (*partial r* = 0.17, *p* <0.001) in normal older adults. Regarding WM tract integrity, we found that BR had negative effects on Fornix in both normal (*partial r* = ‐0.16, *p* = 0.01) and MCI (*partial r* = ‐0.23, *p* = 0.04).

**Conclusion:**

CR has significant positive effects on all cognitive domains but has differential cognitive impacts between normal and MCI older adults. On the other hand, BR has significant negative effects on Fornix in both normal or MCI older adults.

